# Assessment of the risk factors associated with multidrug-resistant tuberculosis in Sudan: a case-control study

**DOI:** 10.4178/epih.e2019014

**Published:** 2019-04-20

**Authors:** Adel Hussein Elduma, Mohammad Ali Mansournia, Abbas Rahimi Foroushani, Hamdan Mustafa Hamdan Ali, Asrar M A/Salam Elegail, Asma Elsony, Kourosh Holakouie-Naieni

**Affiliations:** 1 Department of Epidemiology and Biostatistics, School of Public Health, Tehran University of Medical Sciences-International Campus, Tehran, Iran; 2 Department of Epidemiology and Biostatistics, School of Public Health, Tehran University of Medical Sciences, Tehran, Iran; 3 Multidrug-Resistant Tuberculosis Unit, Communicable and Non-Communicable Diseases Control Directorate, Ministry of Health, Khartoum, Sudan; 4 National Tuberculosis Reference Laboratory, National Public Health Laboratory, Ministry of Health, Khartoum, Sudan; 5 The Epidemiological Laboratory (Epi-Lab), Khartoum, Sudan

**Keywords:** Risk factors, Tuberculosis, Multidrug resistant, Sudan

## Abstract

**OBJECTIVES:**

The emergence of multidrug-resistant tuberculosis (MDR-TB) is a major challenge for the global control of tuberculosis (TB). The aim of this study was to determine the risk factors associated with MDR-TB in Sudan.

**METHODS:**

This case-control study was conducted from May 2017 to February 2019. Patients newly diagnosed with MDR-TB were selected as cases, and controls were selected from TB patients who responded to first-line anti-TB drugs. A questionnaire was designed and used to collect data from study participants. Logistic regression was used to evaluate associations between risk factors and MDR-TB infection. The best multivariate model was selected based on the likelihood ratio test.

**RESULTS:**

A total of 430 cases and 860 controls were selected for this study. A history of previous TB treatment (adjusted odds ratio [aOR], 54.85; 95% confidence interval [CI], 30.48 to 98.69) was strongly associated with MDR-TB infection. We identified interruption of TB treatment (aOR, 7.62; 95% CI, 3.16 to 18.34), contact with MDR-TB patients (aOR, 5.40; 95% CI, 2.69 to 10.74), lower body weight (aOR, 0.89; 95% CI, 0.87 to 0.91), and water pipe smoking (aOR, 3.23; 95% CI, 1.73 to 6.04) as factors associated with MDR-TB infection.

**CONCLUSIONS:**

Previous TB treatment and interruption of TB treatment were found to be the main predictors of MDR-TB. Additionally, this study found that contact with MDR-TB patients and water pipe smoking were associated with MDR-TB infection in Sudan. More efforts are required to decrease the rate of treatment interruption, to strengthen patients’ adherence to treatment, and to reduce contact with MDR-TB patients.

## INTRODUCTION

Tuberculosis (TB) continues to infect millions of people each year, representing a major health challenge and constituting one of the main causes of death worldwide. Approximately 10 million of TB new cases were reported, and 1.3 million deaths occurannually due to TB infections, the majority of which are in resource limited countries [1]. Multidrug-resistant tuberculosis (MDR-TB) is defined as resistance to at least isoniazid and rifampicin, with or without resistance to other anti-TB drugs. The prevalence of MDR-TB is high in sub-Saharan Africa, particularly among patients who have a history of previous TB treatment [2]. In 2017, it was estimated that 558,000 new cases of MDR-TB occurred worldwide, and that 230,000 deaths were due to infections with MDR-TB [3].

Interrupting treatment with anti-TB medicines in an individual infected with TB allows some bacteria to remain alive, giving them a chance to develop resistance. MDR-TB can either occur due to inadequate treatment or direct contact with a MDR-TB patient [4]. Furthermore, MDR-TB is considered to be a main barrier to the control of TB in humans worldwide [5]. Numerous risk factors for the development of TB resistance have been established by the World Health Organization [6]. Many studies have identified risk factors associated with MDR-TB, including poor adherence to treatment, improper dosage, a short duration of treatment, and inadequate drugs [7].

A case-control study conducted in Ethiopia identified a history of TB treatment as a main determinant of MDR-TB [8]. Additionally, numerous studies conducted in Europe reported that a history of previous TB treatment was a main predictor of MDR-TB [9]. In another case-control study performed to identify the determinants of MDR-TB in patients who underwent first-line treatment in Addis Ababa, Ethiopia, interruption of treatment for at least a day and a duration of treatment between 2 months and 7 months were significantly associated with the occurrence of MDR-TB [10]. In a study conducted in China, the following MDR-TB risk factors were identified: previous TB treatment, a high school or lower education, a long distance between the patient’s residence and the health facility, smoking, and adverse effects of anti-TB medication (adjusted odds ratio [aOR], 2.39; 95% confidence interval [CI], 1.40 to 3.26) [11].

In Sudan, the incidence of TB is 77 per 100,000, including TB in human immunodeficiency virus (HIV)-positive individuals, and the total number of new and relapsed TB cases was 20,438 in 2017. The estimated number of MDR-TB cases among notified pulmonary TB cases was 600. The estimated proportion of MDR-TB cases among previously treated patients was 13.0%, while that among patients newly diagnosed with TB was 2.9% [12]. Although many studies have investigated the prevalence and molecular characteristics of MDR-TB in Sudan, few studies have explored risk factors associated with MDR-TB. In a study conducted in Kassala, eastern Sudan from 2007 to 2009, an analysis of 53 isolates from pulmonary TB patients indicated that the prevalence of multidrug resistance was 9.4% [13]. In another study conducted to estimate multidrug resistance among TB patients in Sudan in 2016, the prevalence of resistance to at least rifampicin and isoniazid was 38.0% using drug-susceptibility testing and 37.3% using line probe assays [14]. In contrast, the prevalence of MDR-TB in Sudan has been reported to be 19.0% among retreated patients and 1.8% among new cases [15]. To the best of our knowledge, this is the first case-control study conducted to estimate the predictors of MDR-TB in Sudan. The aim of this study was to identify risk factors leading to the occurrence of MDR-TB using a case-control study design.

## MATERIALS AND METHODS

### Study population and design

The study population consisted of MDR-TB patients (cases) and TB patients who were susceptible to anti-TB drugs (controls). The cases were recruited from MDR-TB treatment units at hospitals and health facilities in various states in Sudan. In total, 29 hospitals and health facilities were sampled to select cases. New patients were included when they were diagnosed and presented to receive treatment.

### Sample size calculation

The sample size was calculated according to the 2-proportion equation, where the null hypothesis was that proportion 1 (p1) equals proportion 2 (p2). The equation for the sample size calculation was n=([r+1]/2)*(Za/2+Zb)^2*^p(1-p)/(p1-p2)^2^; where p= (p1+p2)/2 (p1 is the exposure among cases and p2 the exposure among control groups). The exposure of the control group (p2) was calculated using the following equation: p1=p2*OR/(1+p2* [OR-1]), where OR is the odds ratio for exposure in the control group. Alpha (Zα2) was calculated as 0.050 (2-sided), the power (Z_β_) equaled 0.900, and r was defined as the ratio of controls to cases. The OR for exposure among the control group was calculated using a value of 1.9. The probability of exposure in the control group was 7.5%. Therefore, a total of 430 cases and 860 controls were included in this study. Cases were patients diagnosed with MDR-TB, while controls represented TB patients who were sensitive to treatment and classified as cured [10].

MDR-TB patients who provided informed consent and were at least 18 years old were included in the study. The inclusion criteria for cases were as follows: resistance to both rifampin and isoniazid, confirmation of MDR-TB by laboratory results, and administration of MDR-TB treatment. Controls were recruited from patients who were sensitive to TB treatment and were considered to have been cured, as confirmed by a negative laboratory result according to the national TB program guidelines. Extrapulmonary TB cases and patients who died were excluded from the study.

### Data collection

Data were collected from 18 states throughout Sudan, which has a population of 36.1 million according to the 2008 census [16]. A questionnaire was designed and used to collect data from participants who met the inclusion criteria. The study began in May 2017 and finished in February 2019. All new MDR-TB cases were recruited for the study. When each case was recruited, 2 controls were selected. Cases were selected from patients referred to the MDR-TB unit of hospitals and other health facilities. Controls were recruited from the national TB program registry, in which each TB patient has a unique identification number with linkage to all relevant information, including his or her telephone number. Controls were selected from the same localities and states where cases were recruited.

To reduce selection bias, controls were recruited from a population with the same overall patterns of exposure as cases (i.e., the hospitals or health centers from which cases were referred). In addition, culture and molecular diagnostic techniques were used to identify cases, as well as their clinical symptoms. A questionnaire was validated and piloted to reduce the likelihood of information bias.

Face-to-face interviews by trained interviewers were used to collect data from the study participants. The interviews of cases were carried out the MDR-TB units of hospitals and health care centers. Data were randomly collected from TB registry records to construct a control group from the areas where cases were selected. A questionnaire was translated into the local language and interviewers received training on it. The training involved data collection techniques and how to interact with participants during the procedure. The validity of the questionnaire was evaluated by a group of experts, including a chest physician, epidemiologist, and public health specialist.

### Statistical methodology

To maintain high data quality, continuous checks were done during the data collection phase. Data analysis was carried out using Stata version 13 (StataCorp., College Station, TX, USA). The analysis compared various characteristics between cases and controls. A descriptive analysis was done to present the frequencies of cases and controls, and regression analysis was conducted to identify associations between the investigated variables and MDR-TB. Variables were designated for inclusion in the model according to our study objectives and previous findings indicating that they might be predictors of MDR-TB. Additionally, some variables were included in the model because the authors considered them to be relevant determinants of MDR-TB. Univariate analysis was used to find associations between dependent and independent variables. We followed the backward method to select variables in order to build our statistical model. The strategy of removing variables was used to identify the best logistic regression model. First, a simple logistic regression was implemented for each variable using the original raw scale of risk factors. In the process of building the best logistic regression model, a p-value<0.2 was used as a cut-point to include variables in the multivariate models, assuming no interaction at that time. According to our cut-point, we selected variables from the univariate model and entered them into the multiple logistic regression model. Then, we removed variables that lost significance in the multiple logistic regression model. Next, we added each variable that was discarded from the univariate model one-by-one to determine whether any remarkable change occurred when they were added to the multiple logistic regression model. The best model was selected based on the likelihood ratio test.

### Ethics statement

This study was approved by the Ethical Committee of Tehran University of Medical Sciences under the ethical approval code IR, TUMS.SPH.REC.1395.1718. The study was also approved by the National Ethical Committee of the Ministry of Health, Sudan (No. FMOH/rd/1/104). A written informed consent form was provided to participants who were able to read, and their signatures were obtained. For those who were not able to read, the information in the informed consent form was read aloud by the interviewers. The objectives of the study were explained on a separate sheet that was attached with the questionnaire and read or explained to participants unable to read. The interviewers emphasized that the participants had the right not to participate in the study if they did not want to.

## RESULTS

The study included 430 cases and 860 controls, with an overall mean age of 37.3±15.2 years. The mean age of the cases and controls was 35.31±13.17 years and 38.25±16.12 years, respectively. This study included 906 males (300 cases, 606 controls) and 384 females (130 cases, 254 controls). The majority of study participants had a primary or secondary education (27.1% and 27.7%, respectively). Participants with no formal education accounted for 19.5% of the study participants. Table 1 presents details regarding the socio-demographic characteristics of the study participants. In total, 162 (37.7%) cases interrupted first-line TB treatment, while only 18 (2.1%) controls did so. The majority of cases and controls responded to TB treatment during the intensive phase (71.2% of cases and 79.4% of controls). Feeling better during first-line TB treatment was the most common reason for interrupting TB treatment. More details regarding demographic and clinical characteristics are shown in Table 1. Variables that showed significant associations in the univariate model were included in the multivariate model (Table 2).

According to the backward elimination procedure, the final model included the following variables: interruption of TB treatment, age group, level of education, weight, contact with MDR-TB patients, water pipe smoking, and the number of family members. The multiple logistic regression model indicated a strong association between a previous history of TB treatment and infection with MDR-TB (aOR, 54.85; 95% CI, 30.48 to 98.69). There was also a powerful statistical association between interrupting TB treatment and MDR-TB (aOR, 7.62; 95% CI, 3.16 to 18.34). The analysis indicated that a higher body weight had a negative association with MDR-TB infection (aOR, 0.89; 95% CI, 0.87 to 0.91). Furthermore, the 25-44 and 45-64 age groups had higher rates of MDR-TB infections (aOR, 3.86; 95% CI, 2.30 to 6.48 and aOR, 4.29; 95% CI, 2.13 to 8.28, respectively). Contact with MDR-TB patients was directly associated with MDR-TB (aOR, 5.14; 95% CI, 2.46 to 10.75) (Table 2). An association was observed between internal migration and infection with MDR-TB (aOR, 3.88; 95% CI, 1.37 to 10.96). Moreover, the aOR for water pipe smokers compared with non-water pipe smokers was 3.23 (95% CI, 1.73 to 6.04). The risk of acquiring MDR-TB among females was twice as high as among males (Table 2).

## DISCUSSION

This study was conducted to identify risk factors associated with MDR-TB in Sudan. The main risk factors for MDR-TB are known to be a previous history of TB treatment and interruption of first-line treatment. This study revealed a strong association between these factors and the occurrence of MDR-TB. In our study, most of the MDR-TB patients had a history of previous TB treatment (67.9%). A similar result was reported in a study conducted in South Africa, in which a history of TB treatment failure was a main predictor of MDR-TB [17,18]. Furthermore, this finding is in accordance with a study conducted in Bangladesh, in which the parentage of MDR-TB patients with a history of TB treatment was higher than that obtained in our study (98.2%) [19]. Studies conducted in Ethiopia reported that previous treatment with anti-TB drugs was the main risk factor for the occurrence of MDR-TB [20,21]. A systematic review carried out in Europe likewise revealed that previous TB treatment was a main risk factor for the development of MDR-TB. The high percentage of treatment interruption indicated that loss to follow-up was common, leading to poor treatment adherence [22]. A study conducted in the Gezira area of central Sudan found that a history of previous TB treatment was associated with MDR-TB [23].

Treatment of MDR-TB is an essential part of the global control of TB. Interruption of first-line anti-TB treatment can be considered the main independent variable in this study, and a high rate of treatment interruption was observed. This finding is in accordance with those of a study conducted in Serbia, in which interruption of TB treatment showed a statistically significant relationship with MDR-TB occurrence. Likewise, another study conducted in Bangladesh reported that TB patients who interrupted treatment had a 4 times higher chance of becoming infected with MDR-TB. According to our findings, the majority of patients who discontinued treatment mentioned that they felt better during the course of treatment. According to the patients statement, other reasons for treatment interruption were included a long treatment period, a long distance to health centers, traveling to a place other than their permanent residence for treatment, and adverse effects. Therefore, it is important to implement community awareness initiatives among TB patients with the goal of promoting the continuation of TB treatment. In addition, TB patients living in remote area, particularly in rural settings, should receive special consideration and targeted treatment. Health services must be expanded throughout Sudan, including all localities and administrative units in various states, with a focus on areas with a high TB burden [4,24-26]. In this study, the chances of becoming infected with MDR-TB were 5 times higher among people who had direct contact with MDR-TB patients than among those who did not. This finding in accordance with a study conducted in Amhara region, Ethiopia, in which contact with MDR-TB patients was found to be among the main predictors of MDR-TB occurrence. Therefore, raising awareness among families of MDR-TB patients is urgently required, and doing so will eventually lead to a decrease in infections [27]. Contact tracing strategies should be strengthened in order to screen all contacts of MDR-TB patients.

In this study, the 25-44 and 45-64 age groups were more likely to be infected with MDR-TB than the other age groups (18-24 years and 65+ years). A case-control study conducted in Bangladesh confirmed this finding in the 25-44 age group, among whom MDR-TB was significantly more common [28]. Numerous studies have reported that females had a higher risk than males for MDR-TB, but our study did not find any significant difference between the sexes in this regard [29,30].

Although smoking has been reported to be a major risk factor for MDR-TB in many studies, our study found no significant association between cigarette smoking and MDR-TB occurrence. On the contrary, water pipe smoking was a statistically significant predictor of MDR-TB. People who smoked water pipe were twice as likely to be infected with MDR-TB than those who did not [31]. Among the study subjects, response to treatment during the intensive phase was twice as common as response to treatment during the continuation phase. Being in prison, cigarette smoking, and travel abroad were not predictors of MDR-TB in the multivariate model. Previous studies have concluded that the link between prison and MDR-TB is not well established [32-34]. Contrary to our study, substantial evidence has been found for an association between tobacco smoking and MDR-TB [35].

Both treatment and prevention of MDR-TB are essential, and require political commitment, quality-assured TB bacteriology, uninterrupted quality-assured drug supply, and a suitable recording and reporting system. Molecular epidemiological studies are required to establish which specific drugs an organism is resistant to before starting anti-TB drugs. A key factor in controlling MDR-TB is the availability of drug-susceptibility tests and trained personnel in clinics and hospitals. More efforts are required to fill gaps in these essential components in Sudan. Our study found that a considerable number of MDR-TB cases were reported among internally displaced persons (IDPs), particularly in the Darfur region, which was recently affected by a civil conflict. This conflict resulted in a humanitarian crisis, causing around 1.9 million people to leave their villages and towns and live as IDPs [36]. Living in such conditions is considered to be a major cause of the low notification rate for both TB in general and MDR-TB in particular. A recent study of TB in conflict areas in Sudan reported a low rate of TB case notification in these areas, as well as a high rate of loss to follow-up [37].

Although HIV infection has been considered to be a main risk factor for MDR-TB in various previous studies, we did not find any association between HIV infection and MDR-TB. This is likely because Sudan has a low prevalence of HIV (0.2%) [38]. In contrast, in study conducted in South Africa, no significant association between HIV infection and MDR-TB was found [39]. Also, a study conducted in Ethiopia indicated that HIV was an independent predictor of MDR-TB. [21]. The results of our study found that a high level of education was a predictor of the development of MDR-TB. Consistent with our findings, another study conducted in Myanmar showed that a high education level was associated with MDR-TB [40]. In a study conducted in Serbia, although MDR-TB was 1.5 times as common among people with a high level of education than among those with a low level of education, this difference was not statistically significant. This trend was similar to that observed in our study, with the difference that in our study, the association reached statistical significance [24]. A study conducted in China reported the opposite results, finding that a primary school or lower level of formal education was a main predictor of MDR-TB [41].

There are several limitations of this study. First, MDR-TB is a severe disease, and some patients with MDR-TB, particularly those living in remote areas, die before diagnosis or treatment initiation, which might have led to selection bias. Another limitation is that cases may overestimate their exposure. This can lead to recall bias, which may bias the association either toward or away from the null hypothesis. Furthermore, controls might provide less information regarding their exposure because they do not have the disease. To reduce this potential source of bias, we selected cases and controls based on laboratory tests of drug susceptibility. Another limitation was that the mean age of the control group was higher than that of the case group. This limitation was addressed in the analysis by dividing participants into age groups and including those groups in the multivariate model. It worth noting in this context that age group was included in the best final model of MDR-TB predictors.

MDR-TB is stigmatized, and people with MDR-TB can be hesitant to acknowledge their status. This stigma posed some difficulties in collecting data, and we therefore collaborated with treatment supporters to tackle those challenges. Since we selected controls from the TB treatment center registry, some controls did not have full contact information. To overcome this problem, we compared this information with the state-level registries. Another limitation of this study is that some participants, particularly older people, had low levels of education and hearing/vision problems, which may have prevented them from filling out data collection tools properly. We overcame this problem by asking patients’ caregivers to help.

In this study, we estimated the risk factors associated with MDR-TB in Sudan. The most important factors associated with MDR-TB infection were interruption of TB treatment, previous history of TB treatment, contact with MDR-TB patients, and being between 25 years and 60 years of age. It is vitally important that TB authorities work to reduce the treatment discontinuation rate and to enhance treatment support for TB patients. More attention is needed to raise awareness of TB transmission, especially among MDR-TB patients’ family members, as well as the community as a whole. Further studies are recommended to address the risk factors associated with MDR-TB in Sudan. Such studies could include cohort studies to assess the risk factors contributing to the development of MDR-TB. In addition, qualitative research is highly recommended to understand the reasons for treatment interruption.

## Figures and Tables

**Table 1.** Demographic and clinical characteristics of the study participants

Characteristics	Cases (n=430)	Controls (n=860)	Total (n=1,290)	p-value
Age (yr)				
Mean±SD	35.31±13.17	38.25±16.12		0.001
Median	32.00	34.00		
Sex				
Male	300 (69.7)	606 (70.4)	906 (70.2)	0.796
Female	130 (30.3)	254 (29.6)	384 (29.7)	
Marital status				
Unmarried	167 (38.8)	328 (38.1)	495 (38.4)	0.019
Married	241 (56.0)	494 (57.4)	735 (56.9)	
Divorce	12 (2.8)	9 (1.0)	21 (1.6)	
Widow	8 (1.9)	29 (3.4)	37 (2.9)	
Separated	2 (0.5)	0 (0.0)	2 (0.2)	
Education level				
Without education	60 (14.1)	192 (22.3)	252 (19.5)	<0.001
*Khalwa*	39 (9.2)	55 (6.4)	94 (7.3)	
Primary	129 (29.9)	220 (25.6)	349 (27.1)	
Secondary	117 (26.8)	240 (27.9)	357 (27.7)	
Diploma	8 (1.9)	1 (0.1)	9 (0.7)	
University or postgraduate	77 (18.1)	152 (17.7)	229 (17.7)	
Meals (per day)				
One	5 (1.2)	0 (0.0)	5 (0.4)	<0.001
Two	85 (19.7)	111 (12.9)	196 (15.2)	
Three	322 (74.9)	743 (86.4)	1,065 (82.5)	
More than three	18 (4.2)	6 (0.7)	24 (1.9)	
Means of transport				
By foot	6 (1.4)	1 (0.1)	7 (0.5)	0.001
Public transport	403 (93.7)	842 (97.9)	1,245 (96.5)	
Private car	20 (4.7)	16 (1.9)	36 (2.8)	
Motorcycle	1 (0.2)	1 (0.1)	2 (0.2)	
Time to reach health center (hr)				
Mean±SD	2.2±1.4	1.9±1.2		0.151
Median	1.2	1.5		
No. of family members (per room)				
Mean±SD	7.5±3.6	8.3±3.6		<0.001
Median	7.0	8.0		
Recent migration (within 6 mo)				
Yes	38 (8.8)	15 (1.7)	53 (4.1)	0.022
No	392 (91.2)	845 (98.3)	1,237 (95.9)	
Permanent migration from birthplace				
Yes	144 (33.5)	235 (27.3)	379 (29.4)	<0.001
No	286 (66.5)	625 (72.7)	911 (70.6)	
Contact with TB patients				
Yes	97 (22.6)	58 (6.8)	155 (12.0)	<0.001
No	309 (71.9)	783 (91.0)	1,092 (84.7)	
I don’t remember	24 (5.5)	19 (2.2)	43 ( 3.3)	
Contact with MDR-TB patients				
Yes	64 (14.9)	2 (0.2)	66 (5.1)	<0.001
No	339 (78.8)	844 (98.2)	1,183 (91.7)	
I don’t remember	27 (6.3)	14 (1.6)	41 (3.2)	
Weight				
Mean±SD	51.3±10.1	63.0±13.5		<0.001
Median	50.5	65.0		
History of previous treatment				
Yes	292 (67.9)	26 (3.0)	318 (24.7)	<0.001
No	138 (32.1)	834 (97.0)	972 (75.3)	
Interruption of TB treatment				
Yes	162 (37.7)	18 (2.1)	180 (14.0)	<0.001
No	268 (62.3)	842 (97.9)	1,110 (86.0)	
Diabetes				
Yes	30 (7.0)	46 (5.3)	76 (5.9)	0.242
No	400 (93.0)	814 (94.7)	1,214 (94.1)	
Fever				
Yes	242 (56.3)	379 (55.9)	723 (54.0)	0.905
No	188 (43.7)	370 (44.1)	567 (46.0)	
Imprisonment				
Yes	52 (12.1)	32 (3.7)	84 (6.5)	<0.001
No	378 (87.9)	828 (96.3)	1,206 (93.5)	
Travel abroad at least once				
Yes	64 (14.9)	74 (8.6)	138 (10.7)	0.001
No	366 (85.1)	786 (91.4)	1,152 (89.3)	
Response to treatment phase				
Intensive	306 (71.2)	683 (79.4)	989 (76.7)	0.001
Continuation	124 (28.8)	177 (20.6)	301 (23.3)	
HIV				
Positive	4 (0.9)	18 (2.1)	22 (1.7)	0.128
Negative	426 (99.9)	842 (97.9)	1,268 (98.3)	

Values are presented as number (%). SD, standard deviation; TB, tuberculosis; MDR-TB, multidrug-resistant tuberculosis; HIV, human immunodeficiency virus.

**Table 2.** Univariate and multiple logistic regression analysis of MDR-TB predictors

Variables	OR (95% CI)	p-value	aOR (95% CI)	p-value
Age (yr)				
18-24	1.00 (reference)		1.00 (reference)	
25-44	1.36 (1.01, 1.82)	0.040	3.86 (2.30, 6.48)	<0.001
45-64	0.86 (0.60, 1.22)	0.408	4.29 (2.13, 8.28)	<0.001
>64	0.42 (0.23, 0.75)	0.004	2.09 (0.79, 5.59)	0.136
Sex				
Female	1.00 (reference)		1.00 (reference)	
Male	1.06 (0.82, 1.37)	0.796	1.98 (1.26, 3.09)	0.003
Education level				
Without education	1.00 (reference)		1.00 (reference)	
*Khalwa*	2.24 (1.37, 3.74)	0.001	1.91 (0.75, 4.85)	0.174
Primary	1.84 (1.30, 2.69)	0.001	1.51 (0.79, 2.86)	0.213
Secondary	1.53 (1.08, 2.24)	0.017	1.97 (1.03, 3.76)	0.040
Diploma	25.63 (3.13, 208.83)	0.003	20.18 (1.78, 228.58)	0.015
University or postgraduate	1.62 (1.08, 2.41)	0.018	2.33 (1.16, 4.67)	0.017
Time to reach health center	1.00 (0.98, 1.08)	0.187	-	-
Weight	0.92 (0.91, 0.93)	<0.001	0.89 (0.87, 0.91)	<0.001
History of previous TB treatment				
No	1.00 (reference)		1.00 (reference)	
Yes	67.07 (43.72, 105.35)	<0.001	54.85 (30.48, 98.69)	<0.001
Interruption of TB treatment				
No	1.00 (reference)		1.00 (reference)	
Yes	28.20 (17.04, 46.89)	<0.001	7.62 (3.16, 18.34)	<0.001
Contact with MDR-TB patients				
No	1.00 (reference)		1.00 (reference)	
Yes	3.55 (2.30, 5.64)	<0.001	5.14 (2.46, 10.75)	
Contact with TB patients				
No	1.00 (reference)		1.00 (reference)	
Yes	2.33 (1.71, 3.17)	<0.001	1.64 (0.96, 2.80)	
Water pipe smoking				
No	1.00 (reference)		1.00 (reference)	
Yes	4.24 (2.96, 6.06)	<0.001	3.23 (1.73, 6.04)	<0.001
Cigarette smoking				
No	1.00 (reference)		-	-
Yes	2.33 (1.76, 3.01)	<0.001	-	-
No. of family members				
No	1.00 (reference)		1.00 (reference)	-
Yes	0.93 (0.87, 0.97)	<0.001	0.92 (0.87, 0.97)	0.002
Internal migration				
No	-	-	1.00 (reference)	
Yes	-	-	3.88 (1.37, 10.96)	0.010
Imprisonment				
No	1.00 (reference)		-	-
Yes	3.56 (2.25, 5.62)	<0.001	-	-
Travel abroad				
No	1.00 (reference)		-	-
Yes	1.85 (1.29, 2.65)	0.001	-	-
IDPs				
No	1.00 (reference)		-	-
Yes	2.03 (0.93, 4.42)	0.074	-	-
Response to treatment				
Continuation phase	1.00 (reference)		-	-
Intensive phase	1.50 (1.19, 2.04)	0.001	-	-
HIV status				
No	1.00 (reference)		-	-
Yes	0.44 (0.14, 1.30)	0.139	-	-
Recent migration (within 6 mo)				
No	1.00 (reference)		-	-
Yes	5.46 (2.96, 10.04)	<0.001	-	-
Means of transport to reach TB treatment center				
By foot	1.00 (reference)		-	-
Public transport	0.07 (0.01, 0.66)	0.019	-	-
Private car	0.22 (0.02, 1.91)	0.165	-	-
Motorcycle	0.16 (0.72, 49.83)	0.097	-	-

MDR-TB, multidrug-resistant tuberculosis; OR, odds ratio; CI, confidence interval; aOR, adjusted odds ratio; TB, tuberculosis; IDP, internally displaced person; HIV, human immunodeficiency virus.
